# The Diagnosis of the Internal Secretory Disorders

**Published:** 1916-08

**Authors:** Henry R. Harrower

**Affiliations:** M. D., F. R. S. M. (Lond.), Los Angeles, California


THE
### TEXAS MEDICAL JOURNAL
Dr. F. E. Daniel, Founder. Established July, 1885

MRS. F. E. DANIEL, - Publisher and Managing Editor

Published Monthly.—Subscription, $1.00 a Year.

VOL. XXXII AUSTIN, AUGUST, 1916 No. 2

The publisher is not responsible for views of contributors

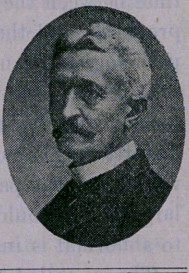

### Original Articles.

### The Diagnosis of the Internal Secretory Disorders.

BY HENRY R. HARROWER, M. D., F. R. S. M. (LOND.), LOS ANGELES, CALIFORNIA.

### IV.

### THE MORE SERIOUS ORGANIC THYROID DISEASES.

Like most organs of the body, the thyroid gland may be the subject of both hypertrophic and degenerative diseases. These exert a well marked influence upon the physiological activities of the organism, not merely due to the effects of the disease *per se*, but more particularly because of the widespread effects of the resulting dyshormonism.

#### THYROID TUMORS—GOITER.

The usual organic disorders such as carcinoma and sarcoma are somewhat rarely found in the thyroid, though this gland is considerably more frequently the seat of a tumor growth known as an adenoma, which may or may not become malignant. It should be understood that these tumors may be present with no decided change in the internal secretory capacity of the gland, while on the other hand either a reduced or an increased functional activity may accompany the evolution of the new growth.

Quite the most common tumors of the thyroid gland are the well known goiters, the incidence and frequency of which is well recognized, and the symptoms of which may vary from the seriously toxic exophthalmic form of goiter to the practically inactive hyperplastic condition. It is very necessary to be able to differentiate between the various forms of thyroid enlargement, since both prognosis and therapeutics depends altogether upon the variety of pathological change that may be present. It must be remembered that at the beginning of puberty and during pregnancy there is a normal functional increase in the work of the thyroid and frequently this is accompanied by a slight enlargement of the gland which has no pathological significance. These physiological enlargements should be watched, however, as the change from normal to abnormal is insidious, and, unfortunately, quite common.

The so-called "simple goiter" may be of three distinct types: (a) Parenchymatous (increased proliferation of the thyroid structure and follicles), in which case the gland is moderately firm to the touch and regular; (b) colloid (increased production of the material in the follicles) with a comparatively large and soft tumor; and (c) cystic (a modification similar to the colloid form in which the follicular contents are fluid) in which there is a distinct fluctuation present.

With these several forms of simple goiter there are often no important general symptoms, although one must expect to find local disturbances depending upon the degree of pressure that may be exerted by the enlarged gland. It can be readily understood that the intrathyroid changes may diminish the secretory powers of the glandular tissue, whilst the hyperplasia causes a considerable increase in the size of the gland. This explains the presence of hypothyroidism in goiter and also the occasional value of thyroid therapy in goiter, for as Falta says, "for the most part there is sufficient parenchyma capable of functionating." When this comparatively healthy portion of the gland is not enough to supply the necessary amount of hormones, the homostimulant action of the thyroid which may be administered, suffices to increase the functional activity of the healthy remainders and thus augment the deficiency with resulting clinical benefit.

The "simple" goiters may and quite frequently do lose their simplicity—the sound secretory portions of the gland may hypertrophy and a condition of hyper-secretion supervene—in which event the clinical diagnosis is not usually made by the local examination, but rather by the study of the manifestations of dyshormonism which accompany the goiter, and which will be referred to shortly.

The well defined and localized goiters are generally called adenomata, and these commonly have a distinctly nodular feeling. Where there is an accompanying syndrome, which includes anemia and cachexia, suspicions of malignancy are warranted. The confirmation of such suspicions is usually made after an operation, although occasionally the presence of metastatic growths is convincing though belated evidence of the malignant character of the tumor.

The thyroid gland may be the subject of an acute infectious process of varying severity. This has been seen to follow an infective process elsewhere in the body, particularly in the tonsils, as well as a number of the acute infectious diseases. Not infrequently it occurs in the primary stage of syphilis. From a diagnostic standpoint the most important findings in acute thyroiditis included the rapid onset and well defined enlargement of the gland with extreme local tenderness, exquisite pain extending up and out to the throat, ears and neck, fever, the results of increased thyroid function—especially cardiac, and, in advanced cases, pus formation with fluctuation.

Sclerotic changes may follow an acute inflammatory process and are not uncommonly also seen in tuberculosis, syphilis and alcoholism. The direct result of this condition is likely to be a varying degree of hypothyroidism (discussed in the previous chapter) which even may become a well defined myxedema.

### MYXEDEMA.

The organic changes in the thyroid just mentioned are less frequent than those functional-organic changes which differ from the minor thyroid disorders outlined in the previous article only in degree. The well defined and chronic secretory disturbances of the thyroid gland are practically always accompanied by structural changes, insufficiency being associated with sclerosis or atrophy and increased activity with hypertrophy and increased vascular engorgement.

The first of these is myxedema or organic hypothyroidism. We have already seen that this may be of very slight degree with a large series of inconspicuous symptoms. It may be more marked —the *myxédème fruste* of Hertoghe, or, again, it may be so well established that the thyroid aplasia results in a typical myxedema, the symptomatology of which we now may discuss briefly. Incidentally the disorder known as cretinism is really an early myxedema or athyroidia, and save for the well defined developmental disturbances due to the earlier lack of the thyroid hormones, the symptoms are practically the same.

Naturally one would expect to find a similarity between the manifestations of myxedema and the minor form of hypothroidism, and this is the case, the difference being chiefly in degree. The changes in the skin are most obvious, and it is due to their prominence that the disease received its name. They are dependent upon the condition of infiltration or edema (which is not really edema, for the infiltrated products are mucoid rather than fluid, and there is no pitting on pressure) which causes well marked trophic changes in the skin itself, as well as in the dermal appendages. The color of the skin is a buff-pink, sometimes almost grayish. It is said by some to look like alabaster. It is puffy, dry, desquamates easily, and the sweat glands are inactive. The skin is often unusually susceptible to local infections. The hair is dry and brittle and falls out in large quantities. The nails crack easily and are dry and poorly nourished. The teeth are almost invariably in very bad order.

The vital processes as a whole are reduced to a minimum. The temperature is from one to several degrees below normal, metabolism is reduced and with it the elimination of wastes by all channels. Toxemia is, therefore, the rule and this favors a condition of invincible constipation which is also usually present. Despite this toxemia the heart action is usually reduced with a slow pulse and a tension often below normal. As a further result of this there is a well marked anemia and especially a hemoglobinemia.

The retrograde changes in the mental powers are very marked, in fact, the whole of the nervous system is extremely inactive. The reaction of the body to external stimuli is very poor. Mentality may vary from dullness to complete amentia, and in early cases loss of memory and inability to concentrate and the general disinclination to use the mental powers is the rule.

Impotence is the rule in men, and in women either amenorrhea or menorrhagia.. (In the first instance the gonads lack the stimuli from the thyroid, which are undoubtedly a factor in establishment and maintaining the molimena, while in the latter the infiltration of the myometrium and endometrium coupled with a subtle change in the chemistry of the blood may cause an increased and prolonged menstrual flow.) Atrophy of the genitalia may take place, but it is not so marked as in hypophyseal disease, of which more later.

### CRETINISM.

In infantile myxedema or sporadic cretinism, in addition to the findings previously mentioned, there is an almost entirely retarded mentality and physical backwardness, and of course the sexual development is practically stopped. The face has a broad, puffy, "sloppy" appearance, the "saddle nose" is frequent, and the capacity to respond by a smile, a twinkle of the eye, or motions of the facial muscles, is almost entirely lost—but this is mental as suitable tests will show no paralysis present. The bones are abnormally formed, short and stubby. The figure is deformed, the gait awkward, and sometimes walking is impossible. Co-ordination is poor. The abdomen is soft and prolapsed, the condition known as "pot-belly" being frequent.

The general metabolic inactivity favors the deposit of fat and the condition of thyroid obesity is quite common in cretinism as in the minor hypothyroid insufficiencies.

A word should be added here about "endemic" cretinism as compared with the "sporadic" form just mentioned. This condition is extremely rare in the United States but common in Switzerland and Austria. The distinction lies in the heredity—endemic cretins are descended from cretin families and are born in places where cretinism is prevalent. The clinical manifestations are, perhaps, not always so completely typical as in the sporadic form, and occasionally procreation is possible. In addition to the stigmata of cretinism already outlined, umbilical hernia are very common in the endemic form of cretinism. Deafmutism is very often associated with thyroid aplasia. According to Scholz nearly 30 per cent of the endemic cretins seen by him were deaf mutes.

There is another important distinction between these two forms of cretinism—the sporadic form responds wonderfully to thyroid medication, while the endemic form may or may not be benefited by this method of treatment.

### HYPERTHYROIDISM.

The other form of thyroid dyscrasia is thyrotoxicosis or hyperthyroidism, and is the best known and most complex of all the functional thyroid diseases. Here the thyroid gland is unusually active with or without a marked increase in its size. This condition is most commonly called "exophthalmic goiter," though an excessive thyroid secretion may be present without the exophthalmos, and, rarely, the exophthalmos may be present without the goiter. (Parenthetically the use of a physician's name to identify this disease is confusing. Parry discovered the syndrome first (1786). Flajani described it again later. Graves explained the syndrome intelligently (1835), while V. Basedow (1843) gave a better description and connected the disorder more definitely with its real cause.)

A few words as to the principal causes of this complex disease may facilitate our study of its diagnosis. Three great factors must be taken into consideration: Focal infections; fright and emotional affections and the thyroid instability so well emphasized by Leopold Levi and discussed previously.

Fright and excessive emotions are not uncommonly connected with the onset of a severe degree of exophthalmic goiter, and Cannon's recent researches into the relation of the emotions to adrenal excitation may be the basis of a satisfactory explanation as to how this is caused. For instance, it is quite possible that the undue stimulation of the adrenals thus brought about may so decidedly push the thyroid pendulum as to cause it to swing very much more rapidly and widely than is normally the case, while the resulting dyshormonism may prolong the effects, for the thyroid itself is just as susceptible to the thyroid hormones in the blood as are any of the other organs of the body.

Toxemia, usually of bacterial origin, is probably the most common cause of this disease, and a careful study very often will reveal some focus of infection in one part of the body or another. Most common among these sources of bacterial poisoning are the tonsils, nasal fossae and adjoining sinuses, teeth and gums (and especially around the roots of the teeth), colon (especially the angles), gall bladder and pelvic organs; probably in the order mentioned. Undoubtedly there is also a connection between the incidence of hyperthyroidism and the gonads, especially in women, and the frequency of this disease in women, about 10 to 1, and the common relationship of menstrual disturbances with it, and *vice versa,* are sufficient confirmation of this. That these gonad disorders are usual in individuals with that subtle disorder named *"l'instabilité thyroidienne"* and that an hereditary defective thyroid substratum favors the onset of dysthyroidism, is well borne out by those who are in the habit of making a thorough anamnesis.

Aside from the two symptoms embodied in the name—exophthalmos and goiter—symptoms which we need not dilate upon here, the most obvious diagnostic finding is the serious change in the heart action. Tachycardia is practically the rule, the pulse ranging from 120 to 180 beats per minute. With this is an extreme degree of nervous irritability, a good part of which, in the estimation of the writer, is due to the functional circulatory changes, although there are cases in which these nervous manifestations have nothing to do with the heart. The cardiac excitability—it is often of a heaving, pounding nature—is responsible for the pulsation, not merely of the goiter itself, but of various vessels throughout the body, and this persistent beating in the head, abdomen and especially the throat and neck is a very uncomfortable symptom. The undue strain on the heart often causes dilatation and even incompetency. Some writers mention the auscultation of a murmur over the goiter.

As might be expected, the metabolism is decidedly plus. All the cells are working overtime as a result of the excessive thyroid stimuli, and this is doubtless responsible for the hyperthermia which is quite common in the well marked cases of this disease. It also accounts for the loss of weight (despite the not infrequently increased appetite and intake of food), the increased perspiration, and, probably, for the sharpened mental activities. In this connection one of the difficult features of hyperthroidism is the control of the mental status with its disturbances of concentration in work and its effects upon insomnia. It has been remarked by several writers, and especially by Leonard Williams of London, that among the earliest signs of excessive thyroid action is a tendency to genius, such individuals have great ideas, lean to literature or the arts, take up fads, and are far from dull in their studies or their work.

With the decided effects of myxedema upon the skin, one would expect some opposite changes in the skin in this opposite condition, and this is the case. The skin is usually thin and delicate, is moist and well nourished by a very good blood supply. The skin reddens under the slightest local or emotional influence and the sweat glands become active on the slightest provocation until the hyperidrosis is more than a nuisance. Occasionally this is one of the earliest symptoms and night sweats of thyroid origin have led the diagnostic scent away from the right trail.

The toxemia, sympathetic irritability and cardio-vascular excitability together form a combination which may produce many and varied symptoms only a few of which need be enumerated, i. e., tremor, twitchings of the eyelids and face, restlessness, insomnia and often a decided neurasthenia. Myasthenia with an aggravating fatigue and much discomfort on exertion are usual and to be expected in a disease in which the hormonic balance is so thoroughly disorganized.

The late symptoms of exophthalmic goiter include serious heart changes, both in the sounds and the rhythm. Heart failure is a common cause of death from this disease. Dyspnea and severe diarrhea are also ominous signs when found accompanying other signs of hyperthyroidism.

EDITOR'S NOTE.—An opportunity is offered to interested readers to submit clinical questions for answer in an early issue.

The next article will be entitled, "The Adrenal Glands in Health and Disease."

